# Investigation of Sub‐Bandgap Emission and Unexpected n‐Type Behavior in Undoped Polycrystalline CdSe_x_Te_1‐x_

**DOI:** 10.1002/advs.202309264

**Published:** 2024-06-03

**Authors:** Deborah L. McGott, Steven W. Johnston, Chun‐Sheng Jiang, Tuo Liu, Darius Kuciauskas, Stephen Glynn, Matthew O. Reese

**Affiliations:** ^1^ National Renewable Energy Laboratory Golden CO 80401 USA

**Keywords:** cadmium telluride, chlorine, defects, n‐type, Se alloy

## Abstract

Se alloying has enabled significantly higher carrier lifetimes and photocurrents in CdTe solar cells, but these benefits can be highly dependent on CdSe_x_Te_1‐x_ processing. This work evaluates the optoelectronic, chemical, and electronic properties of thick (3 µm) undoped CdSe_x_Te_1‐x_ of uniform composition and varied processing conditions (CdSe_x_Te_1‐x_ evaporation rate, CdCl_2_ anneal, Se content) chosen to reflect various standard device processing conditions. Sub‐bandgap defect emission is observed, which increased as Se content increased and with “GrV‐optimized CdCl_2_” (i.e., CdCl_2_ anneal conditions used for group‐V‐doped devices). Low carrier lifetime is found for GrV‐optimized CdCl_2_, slow CdSe_x_Te_1‐x_ deposition, and low‐Se films. Interestingly, all films (including CdTe control) exhibited n‐type behavior, where electron density increased with Se up to an estimated ≈10^17^ cm^−3^. This behavior appears to originate during the CdCl_2_ anneal, possibly from Se diffusion leading to anion vacancy (e.g., V_Se_, V_Te_) and Cl_Te_ generation.

## Graphical abstract

A high density (≈10^16^ cm^‐3^) of shallow donors is found in “undoped” polycrystalline CdSe_x_Te_1‐x_ processed using conditions reflective of standard devices. This behavior appears to originate during the CdCl_2_ anneal, possibly from generation of anion vacancies (e.g., V_Se_, V_Te_), Cl_Te_, and related complexes via Se diffusion. This may pose serious concerns for group‐V doped CdSe_x_Te_1‐x _devices.

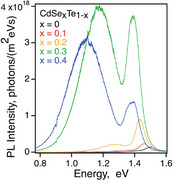

## 1 Introduction

Cadmium telluride (CdTe) photovoltaics (PV) are important to the health of the U.S. PV market, making up ∼40% of the utility‐scale market and ≈25% of plants >1 MW.^[^ ^1^, ^2^ ^]^ Since 2002, device efficiency has improved from 16.7 to 22.6%,^[^ ^3^, ^4^ ^]^ where increases up to 22.1% were largely enabled by Se alloying at the front of the absorber to form CdSe_x_Te_1‐x_ (CST).^[^ ^5^ ^]^ This allowed for bandgap engineering and led to significant boosts in current density, carrier lifetime, and deep‐level defect passivation.^[^ ^6^, ^7^, ^8^, ^9^, ^10^ ^]^ The final 0.5% improvement resulted from a shift in doping chemistry from Cu, which has largely limited absorber hole density to mid 10^14^ cm^−3^, to group V dopants (“GrV”, e.g., As, P), which has enabled carrier concentrations >10^16^ cm^−3^ in polycrystalline devices.^[^ ^11^, ^12^, ^13^ ^]^ As absorber hole density increases, theoretical studies have shown recombination at or near the front interface becomes limiting.^[^ ^14^, ^15^, ^16^ ^]^ Thus, it is increasingly important to not only understand the improvements enabled by Se alloying, but also what losses might originate in the CdSe_x_Te_1‐x_ layer.

This work attempts to isolate Se‐related losses by studying thick (3 µm) evaporated CdSe_0.3_Te_0.7_, the composition used in champion NREL devices, with no intentional doping and processing conditions varied to reflect standard device processing. Conditions evaluated were CdSe_0.3_Te_0.7_ deposition rate (reflective of historically‐used slow growth versus recent faster growth), CdCl_2_ anneal conditions (i.e., optimized for Cu‐ vs GrV‐doped devices), and CdSe_x_Te_1‐x_ composition (reflective of different source materials tested before arriving at CdSe_0.3_Te_0.7_ as the “baseline”). Optoelectronic, chemical, and electrical properties of test structures were characterized using a suite of techniques including photoluminescence (PL), time‐resolved photoluminescence (TRPL), Auger electron spectroscopy (AES), deep‐level transient spectroscopy (DLTS), and scanning‐spreading resistance microscopy (SSRM). Sub‐bandgap defect emission ≈100–200 meV from the exciton peak was observed, which increased relative to the exciton peak as Se content increased and with “GrV‐optimized CdCl_2_” (i.e., CdCl_2_ anneal conditions used for GrV‐doped devices). Lifetime decreased with decreasing Se content; for CdSe_0.3_Te_0.7_, both a slow deposition rate and GrV‐optimized CdCl_2_ dramatically decreased lifetime (from 920 ns to 140–150 ns). The low lifetime in “slow deposition CdSe_0.3_Te_0.7_” is attributed to reduced Se content in the final film (measured CdSe_0.23_Te_0.77_ via AES) with additional losses potentially from anion vacancy (i.e., V_Se_, V_Te_) generation. Low lifetime in CdSe_0.3_Te_0.7_ with GrV‐optimized CdCl_2_ appeared to result from increased nonradiative recombination, possibly from a broader band of defects relative to CdSe_0.3_Te_0.7_ with Cu‐optimized CdCl_2_ (“baseline”).

“Undoped” CdTe and CdSe_x_Te_1‐x_ are typically thought to be slightly p‐type, usually due to Cd vacancies and/or Cu dopants unintentionally introduced during the CdCl_2_ anneal.^[^ ^17^, ^18^ ^]^ All films studied here, however, were found to be n‐type where electron density increased with Se content from barely detectable (CdTe) to ≈10^16^–10^17^ cm^−3^ for CdSe_0.3_Te_0.7_ and CdSe_0.4_Te_0.6_, despite using conditions similar to standard device processing. The strong n‐type behavior in the absence of intentional doping is surprising, particularly since electron density is about the same as the desired hole density in GrV‐doped devices. Throughout the work, we develop the hypothesis that this n‐type behavior, and possibly sub‐bandgap defect emission, originate from the CdCl_2_ anneal. Specifically, anion vacancies may form more readily in CdSe_x_Te_1‐x_ than CdTe,^[^ ^17^ ^]^ which could then be filled with chlorine to form Cl_Te_, a known shallow donor defect in CdTe.^[^ ^19^, ^20^, ^21^ ^]^ Unintentional n‐type behavior in CdSe_x_Te_1‐x_ at the front of devices could result in losses from buried junction effects, compensation, low activation, and so on.

For clarity, characterization results are first presented with minimal analysis in Section 2. After all results are presented, they are analyzed and discussed en masse in Section 3, which is divided into three subsections to highlight and evaluate the significance of the results in their varied aspects. Section 3.1 explores the effect that processing conditions have on performance; Section 3.2 discusses the observed n‐type behavior and its implications in devices; and Section 3.3 takes a deeper dive into possible underlying mechanisms.

## 2 Results

**Figure 1:**
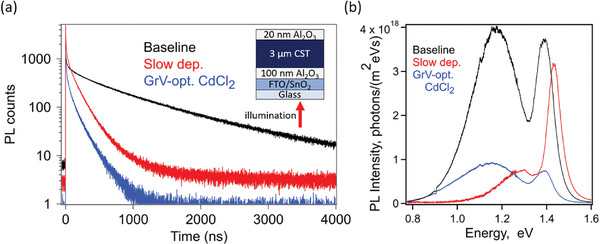
a) TRPL and b) spectrally corrected absolute PL for double heterostructures (stack shown in the inset of panel a) evaporated from an alloyed CdSe_0.3_Te_0.7_ source with “baseline” conditions (16 Å s^−1^ CST deposition, CdCl_2_ anneal developed for Cu‐doped devices – black traces), “slow dep.” (2 Å s^−1^ CST deposition – red traces), and “GrV‐opt. CdCl_2_” (CdCl_2_ anneal conditions optimized for GrV‐doped devices – blue traces).

**Figure** **1** shows PL‐related data for CdSe_0.3_Te_0.7_ double heterostructures (DHs–3 µm thick CdSe_0.3_Te_0.7_ sandwiched between two passivating Al_2_O_3_ layers, film stack shown in Figure 1a inset) with (black trace) “baseline” conditions for high lifetime test structures, i.e., evaporation from a CdSe_0.3_Te_0.7_ alloyed source at 16 Å s^−1^ and CdCl_2_ anneal conditions developed for Cu‐doped devices; (red trace) “slow dep.”, i.e., 2 Å s^−1^ deposition (reflective of historically used processing) rather than 16 Å s^−1^ and same CdCl_2_ anneal; and (blue trace) “GrV‐optimized CdCl_2_,” 16 Å s^−1^ deposition and CdCl_2_ anneal developed for GrV‐doped devices. “Baseline” CdCl_2_ anneal conditions were 500 ˚C for 10 min under 400 Torr He. These are standard processing conditions for test structures as they have been shown to maximize carrier lifetimes,^[^ ^8^ ^]^ but are slightly more “aggressive” than CdCl_2_ anneals typically done in devices (450–480 ˚C), as devices tend to start delaminating at ≈500 ˚C.

Minority carrier lifetime in the bulk^[^ ^14^ ^]^ was calculated by fitting the “tail” of TRPL decays (at long time scales) in Figure 1a using:

(1) $$I\left(t\right)\;=\;I_{0}e^{-t/\tau_{2}}$$

where *I(t)* = intensity, *I_0_* = initial intensity, *t* = time, and τ_2_ = minority carrier lifetime of the tail. τ_2_ values are listed in **Table** **1**. TRPL was collected at wavelengths between about 700 and 1100 nm (energy between ≈1.1 and 1.8 eV) using a 700 nm longpass filter and Si avalanche photodiode detector. While baseline CdSe_0.3_Te_0.7_ showed a high τ_2_ of 920 ns, the “slow deposition” and “GrV‐optimized CdCl_2_” CdSe_0.3_Te_0.7_ only reached 140–150 ns. The absolute PL in Figure 1b shows a large defect peak ≈200 meV below the exciton peak for baseline CdSe_0.3_Te_0.7_ (it is important to note this does not necessarily equate to the thermal activation energy of the defect). Under slow deposition conditions, the defect peak height decreased relative to the exciton peak, and with “GrV‐optimized CdCl_2_,” the overall luminescence decreased but the defect peak grew relative to the exciton peak (see Figure S1, Supporting Information for normalized PL data). Photoluminescence quantum yield (PLQY, integration under the entire PL curve) is listed in Table 1.

**Table 1:** Measured parameters for CdSe_x_Te_1‐x_ evaporated from an alloyed CdSe_0.3_Te_0.7_ source with “baseline” conditions (16 Å s^−1^ deposition, CdCl_2_ anneal developed for Cu‐doped devices), “slow deposition” (2 Å s^−1^ deposition rather than 16 Å s^−1^), or “GrV‐optimized CdCl_2_” conditions (CdCl_2_ anneal developed for GrV‐doped devices). Carrier lifetime (τ_2_) measured via TRPL; energy at exciton and defect peak maxima (*E_exciton_* and *E_defect_*, respectively) and photoluminescence quantum yield (PLQY) obtained from PL; Se/(Se+Te) in the final CdCl_2_‐ treated film measured via AES; average resistance measured using SSRM under −5 V sample bias voltage (*R_tot_*); and grain size measured with bright‐field optical microscope.

	τ_2_ [ns]	E_exciton_ [eV]	E_defect_ [eV]	PLQY	Se/(Se+Te)	R_tot_ [Ω]	Avg. grain size [µm]
**Baseline**	920	1.39	1.17	7.2E‐04	29	5.5E + 04	1.9 ± 0.1
**Slow deposition**	150	1.43	1.30	2.2E‐04	23	3.7E + 05	2.2 ± 0.3
**GrV‐optimized CdCl_2_**	140	1.39	1.16	1.9E‐04	27	4.4E + 04	2.1 ± 0.1

AES was used to measure Se/(Se+Te) ratios (also called Se %, concentration, or content in this work) in the CdCl_2_‐treated CdSe_x_Te_1‐x_ films by cleaving at the Al_2_O_3_/CdSe_x_Te_1‐x_ interface and ion milling (see Experimental Section for details); values are given in Table 1. While “GrV‐optimized CdCl_2_” conditions did not significantly impact Se concentration, reducing the CdSe_0.3_Te_0.7_ deposition rate from 16 to 2 Å s^−1^ caused Se in the film to drop from 29 to 23 at%. This is discussed further in Section 3.1.

**Figure 2:**
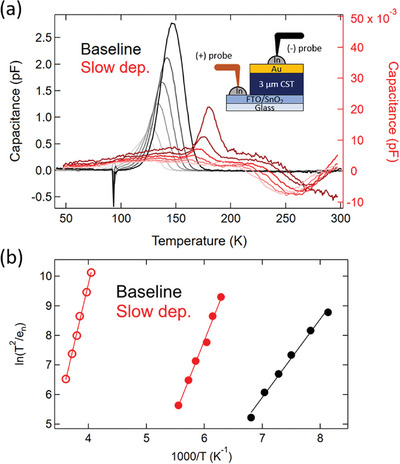
DLTS measurements showing a) capacitance as a function of temperature for varied transient time windows (from lightest to darkest = 100, 50, 20, 10, 5, and 2 ms) and b) Arrhenius plot using the peak temperature values for the corresponding time windows where closed circles represent minority carrier (hole) traps and open circles represent majority carrier (electron) traps. Black tones represent baseline CdSe_0.3_Te_0.7_ and red tones represent “slow deposition CdSe_0.3_Te_0.7_” (measured CdSe_0.23_Te_0.77_). Inset of (a) shows the device stack measured and reversed polarity of probes required for measurement.

SSRM is an atomic force microscopy (AFM)‐based electrical technique used for nm‐scale resistance mapping (typically with a spatial resolution of 10–50 nm, depending on sample and probe conditions).^[^ ^22^ ^]^ While the resistance along the entire current path through the film stack is involved, the SSRM‐measured resistance (*R*_tot_) is dominated by spreading resistance beneath the probe (*R*_sp_, see Experimental Section for discussion), where the probe depth is ≈50 nm. The change in *R*_tot_ as bias voltage is switched from positive to negative polarity can give insight into carrier type. SSRM was measured on CdCl_2_‐treated cleaved CdSe_0.3_Te_0.7_ with Au back contacts; *R_tot_* is listed in Table 1. Interestingly, all films showed n‐type behavior (Figure S2, Supporting Information), which was corroborated by the Hall effect (not shown, done on cleaved CdSe_x_Te_1‐x_ films with no back contact) and the probe polarity required to measure DLTS (reversed from a standard p‐type structure, shown in **Figure** **2**a inset). It was surprising that these films were measurable via Hall since polycrystalline CdTe‐based materials (no intentional doping) are typically below the detection limit of the system used (≈10^16^ cm^−3^).

A rough estimate for electron density can be extracted from *R*_tot_ using:

(2) $$\frac{1}{\rho }=\;qC\mu \;=\;\frac{1}{4rR}$$

where ρ = resistivity, q = elemental charge, C = charge concentration, μ = mobility, r = probe/sample contact radius, and R = measured resistance. Calculated electron density for CdSe_0.3_Te_0.7_ with baseline conditions and “GrV‐optimized CdCl_2_” was ≈10^17^ cm^−3^ and electron density for “slow deposition CdSe_0.3_Te_0.7_” (actually CdSe_0.23_Te_0.77_) was ≈10^16^ cm^−3^, assuming electron mobility in all films is relatively constant. However, it is unclear if this is a fair assumption as mobility may change significantly with Se alloying,^[^ ^23^ ^]^ so electron densities are not given in Table 1. *R*_tot_ was uniform laterally, across grain boundaries (GBs; example in Figure S2b, Supporting Information), and throughout the thickness (Figure S2g, Supporting Information). *R*_tot_ was measured across 12 µm; grain size for all three films was ≈2 µm (see Figure S3, Supporting Information; Table 1).

DLTS is used to measure the transient capacitance change after deep‐level traps in the space charge region are filled with either majority‐ or minority‐carrier charges. Figure 2 shows DLTS results for baseline CdSe_0.3_Te_0.7_ and “slow deposition CdSe_0.3_Te_0.7_” (both CdCl_2_‐treated) with SnO_2_:F/SnO_2_ as the front contact and Au as the back contact. CdSe_0.3_Te_0.7_ with GrV‐optimized CdCl_2_ was not measurable via DLTS or capacitance‐voltage (CV, not shown) and behaved as if contact was not being made. This may indicate an issue with one or both contacts and is discussed in Section 3.1. As mentioned above, probe polarity during DLTS measurement was reversed from what would typically be used for p‐type absorbers (Figure 2a inset). This suggests that the back Schottky barrier was probed rather than the front (typically p‐n but in this case n‐n) heterojunction. Because of this, and due to buried junction effects observed previously in graded CST devices^[^ ^24^ ^]^ which would dominate the capacitance signal at the front, it is not likely that effects such as band offset at the SnO_2_/CST interface have a significant impact on DLTS measurements.

The positive peaks in Figure 2a indicate minority carrier trapping, which in n‐type material are hole traps. Baseline CdSe_0.3_Te_0.7_ has a hole trap while “slow deposition CdSe_0.3_Te_0.7_” (measured CdSe_0.23_Te_0.77_) shows a hole trap and a negative peak corresponding to a majority carrier (electron) trap that forms at higher temperature, giving a defect level deeper in the bandgap. For both samples, peak heights, and widths increased as the transient time window decreased, suggesting nonexponential decay shapes and a band of defects rather than a low concentration of point defects. Figure 2b shows an Arrhenius plot for the two samples from which activation energy (*E_A_*) of the carrier traps, carrier density (*N_S_*), and trap density (*N_T_*) are extracted;^[^ ^25^ ^]^ values are listed in **Table** **2**. Changes in peak height were still observed at the lowest time resolution of the DLTS system, so the calculated *N_T_* is considered a lower bound. It is noted that DLTS showed similarly high electron densities to SSRM. The apparent capture cross‐section (σ_a_) of the hole traps is calculated assuming a hole‐effective mass of 0.63 m_o_ (where m_o_ is electron mass).^[^ ^26^ ^]^ It is noted, however, that this value may hold little physical relevance if there is any temperature dependence in the capture rate.

**Table 2:** Measured DLTS parameters for CdSe_x_Te_1‐x_ evaporated from an alloyed CdSe_0.3_Te_0.7_ source with “baseline” and “slow deposition” conditions (CdSe_0.3_Te_0.7_ with “GrV‐optimized CdCl_2_” could not be measured). Activation energy (*E_A_*), carrier density (*N_S_*), trap density (*N_T_*), and apparent capture cross section (σ_a_) were calculated from the DLTS data. The “slow dep. (electron trap)” *E_A_* is with respect to the conduction band and N_S_ = holes; “baseline” and “slow dep. (hole trap)” *E_A_* is with respect to the valence band and N_S_ = electrons.

	E_A_ [eV]	N_S_ [cm^−3^]	N_T_ [cm^−3^]	σ_a_ [cm^2^]
**Baseline**	0.23 ± 0.01	7.9E + 15	4.8E + 14	7.6E‐17
**Slow dep. (hole trap)**	0.42 ± 0.02	1.6E + 14	4.8E + 12	4.0E‐13
**Slow dep. (electron trap)**	0.70 ± 0.02	1.6E + 14	1.8E + 12	1.3E‐12

**Figure 3:**
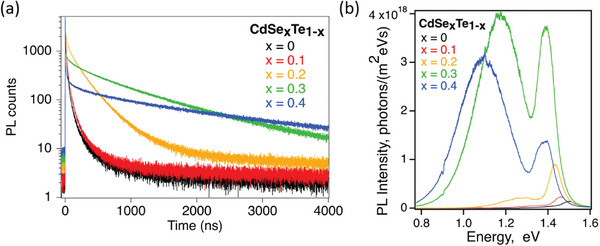
a) TRPL and b) absolute spectrally corrected PL showing the effect of increasing Se concentration in CdSe_x_Te_1‐x_ from x = 0 to 0.4 (referring to the alloyed source material composition) with baseline conditions; x = 0.3 data copied here for reference.

Using baseline conditions (16 Å s^−1^ deposition, CdCl_2_ developed for Cu‐doped devices), Se % was varied by evaporating from alloyed CdSe_x_Te_1‐x_ sources with x = 0 (CdTe), 0.1, 0.2, and 0.4, in addition to the x = 0.3 explored above. This series was done in a more complete sweep since CdSe_x_Te_1‐x_ composition is graded in devices. **Table** **3** lists extracted characterization data similar to Table 1. **Figure** **3** shows TRPL and absolute PL data; normalized PL data is in Figure S4 (Supporting Information). Generally, the measured Se % in the films was close to but slightly below the source material, suggesting a slight loss of Se during deposition and/or during subsequent CdCl_2_ treatment. Se concentration and *E*_exciton_ (often equated with bandgap) measured here agree with literature demonstrating a bandgap “bowing” effect in CdSe_x_Te_1‐x_.^[^ ^27^ ^]^

**Table 3:** Measured parameters for CdSe_x_Te_1‐x_ evaporated from alloyed source powders with x = 0, 0.1, 0.2, 0.3 (copied from above), and 0.4 under baseline conditions (16 Å s deposition, CdCl_2_ developed for Cu‐doped devices). τ_2_ was measured via TRPL; *E_exciton_*, *E_defect_*, and PLQY via PL; Se/(Se+Te) measured in the final films via AES; *R_tot_* measured using SSRM; and grain size measured with bright‐field optical microscope.

	τ_2_ [ns]	E_exciton_ [eV]	E_defect_ [eV]	PLQY	Se/(Se+Te)	R_tot_ [Ω]	Avg. grain size [µm]
**CdTe**	33	1.50	N/A	9.9E‐06	0	1.7E+06	3.2 ± 0.3
**CdSe_0.1_Te_0.9_**	42	1.46	N/A	1.7E‐05	8	9.0E+05	3.4 ± 0.1
**CdSe_0.2_Te_0.8_**	230	1.43	1.28	6.2E‐05	19	1.8E+05	2.3 ± 0.2
**CdSe_0.3_Te_0.7_**	920	1.39	1.17	7.2E‐04	29	5.5E+04	1.9 ± 0.1
**CdSe_0.4_Te_0.6_**	1300	1.38	1.10	5.2E‐04	36	2.6E+04	1.6 ± 0.2

As Se increased, τ_2_ increased (in agreement with previous studies),^[^ ^7^, ^8^ ^]^ defect emission increased (in agreement with previous studies),^[^ ^23^, ^28^, ^29^ ^]^ grain size decreased (Figure S5, Supporting Information, also in agreement with previous studies),^[^ ^30^ ^]^ and *R*_tot_ decreased (i.e., electron density increased). All films, including CdTe, demonstrated n‐type behavior despite using processing conditions standardly used for high‐lifetime test structures^[^ ^8^, ^31^, ^32^ ^]^ and expected to result in slightly p‐type films. The measured resistance decreased by two orders of magnitude when Se content increased from 0% (CdTe) to ≈40%, indicating electron density likely increases by a similar amount assuming mobility stays relatively unchanged. It is noted, however, that CdTe, CdSe_0.1_Te_0.9_, and CdSe_0.2_Te_0.8_ were too resistive to be measured via Hall (with measurement threshold ≈10^16^ cm^−3^) and CV showed decreasing capacitance with voltage for both probe polarities on CdTe, so it is likely only lightly n‐type.

## 3 Discussion

The above results are now discussed in an integrated manner where discussion is divided into three sections. The first section focuses on the effect processing conditions have on CdSe_x_Te_1‐x_ performance, particularly carrier lifetime. The second section discusses the n‐type behavior observed here and its potential implications in devices. The third section explores mechanisms, including Se‐ and Cl‐related defects, which may be responsible.

### 3.1 Processing Parameters Effect on Performance

One of the major benefits of alloying CdTe with Se is the significantly improved carrier lifetime. However, lifetime was shown to be strongly dependent on processing conditions, which can vary widely between institutions. In particular, deposition rate, which is often not even listed in experimental sections, showed a dramatic effect on carrier lifetime when the CdSe_0.3_Te_0.7_ source was evaporated slowly (i.e., sample and source held at high temperature under vacuum for longer). Importantly, AES showed slow deposition resulted in reduced Se % in the final film (CdSe_0.23_Te_0.77_ measured), possibly resulting in a high density of anion vacancies which can act as nonradiative recombination centers, reducing lifetime and luminescence. While the CdTe community has largely not considered preferential loss of Se during high‐temperature deposition of Se‐containing films, it is not unknown. In fact, binary CdSe films are commonly n‐type from high densities of V_Se_,^[^ ^33^, ^34^ ^]^ and evaporation of CuIn_x_Ga_1‐x_Se_2_ is frequently done with a Se overpressure to prevent V_Se_ formation.^[^ ^35^ ^]^

Here, it appears changes in TRPL and PL can mostly be explained by the lower Se content, as the CdSe_0.23_Te_0.77_ (slow deposition) sample closely resembled CdSe_0.19_Te_0.81_ (deposited from a CdSe_0.2_Te_0.8_ source) with regard to grain size, *R*_tot_, τ_2_, and, remarkably, PL curves looked nearly identical when normalized (see Figure S6 and Table S1, Supporting Information for direct comparison between the two). Additional losses in lifetime (i.e., the CdSe_0.23_Te_0.77_ slow deposition sample was 150 ns while CdSe_0.19_Te_0.81_ was 230 ns) may be due to anion vacancies and/or other defect chemistries, as discussed in Section 3.3. This may be reflected in the difference in DLTS between baseline and slow deposition CdSe_0.3_Te_0.7_ seen in Figure 2. It could be that as the density of defects that dominates the sub‐bandgap PL peak and capacitance for baseline CdSe_0.3_Te_0.7_ decrease, a defect(s) deeper in the band becomes visible, or it could be the same defect shifted deeper under “slow deposition” conditions. Further research is required.

An important recent shift in processing for the CdTe community has been from Cu to GrV doping, for which CdCl_2_ anneal conditions have been re‐optimized. Here, CdSe_0.3_Te_0.7_ treated with GrV‐optimized CdCl_2_ conditions showed substantially lower lifetime than baseline (i.e., Cu‐optimized CdCl_2_) CdSe_0.3_Te_0.7_ (140 and 920 ns, respectively). Together with decreased PLQY and broadened defect peak, low lifetime likely results from increased nonradiative recombination and a broader band of defects. Since CST is typically responsible for high lifetimes in Cu‐doped devices, this reduced lifetime with GrV‐optimized CdCl_2_ may be a reason that GrV‐doped devices can suffer from relatively low lifetimes, particularly when absorber hole density is low.^[^ ^18^ ^]^ Unfortunately, DLTS (or CV) could not be measured on these samples; SSRM, however, was measurable. Because DLTS and CV require current flow through both contacts while SSRM only requires an intact back contact (see Experimental Section), this suggests that the SnO_2_/CdSe_0.3_Te_0.7_ interface may be damaged during the “GrV‐optimized CdCl_2_” anneal (i.e., defect density greatly increased – GrV‐dopant pileup is regularly observed in devices and may be related).^[^ ^3^, ^36^ ^]^ In highly doped GrV devices, this issue may be exacerbated by the increased sensitivity to front interface recombination.^[^ ^14^, ^15^, ^16^ ^]^

Finally, the samples with the highest PLQY, which is typically taken as an indication of better material passivation,^[^ ^7^, ^8^, ^37^, ^38^ ^]^ also showed the highest sub‐bandgap emission (e.g., CdSe_0.3_Te_0.7_ and CdSe_0.4_Te_0.6_ in Figure 3b). This raises the question of whether high PLQY (integration under the entire PL curve) always indicates good material quality. Since increasing defect emission with higher Se content has been observed for undoped CST fabricated at other institutions using different methods,^[^ ^28^, ^39^ ^]^ this hints at a fundamental defect that may lead to losses in devices. It is unclear how harmful these defects are though, since Cu‐doped devices with CdSe_0.3_Te_0.7_ at the front still achieve high carrier lifetime and photocurrent.^[^ ^31^, ^40^ ^]^ Because the defects are radiative and relatively shallow, it is possible that the long carrier lifetimes originate from the trapping/de‐trapping of minority carriers (holes).^[^ ^23^ ^]^ This is supported by DLTS, which showed the dominance of hole trapping in these films. TRPL curves do not suggest detrimental trapping though (i.e., nearly complete decay within first few nanoseconds followed by a flat tail hovering just above baseline),^[^ ^41^ ^]^ likely because deep (nonradiative) defects in CdTe are passivated by Se.^[^ ^6^, ^10^ ^]^ Passivation of deep defects and introduction of shallow hole traps at GBs via Se and Cl^[^ ^10^ ^]^ may be a reason that lifetime and conductivity increased as grain size decreased (Figure S7, Supporting Information), both unexpected trends.

Because SSRM did not show a distinguishable difference between GBs and grain interiors (GIs), this suggests that electron density is within a factor of ten between the two. Since the GB region is likely only a few atomic layers thick (less than a few nanometers), it is possible that the resistivity change around the GB is not detected via SSRM, which has a spatial resolution of 10–50 nm. It is noted, however, that changes in GB resistivity have been detected for CdTe and other PV materials using the same SSRM tool previously.^[^ ^42^, ^43^ ^]^ Additionally, a large forward bias (5 V) is applied during SSRM measurement to overcome probe/sample contact resistance (see Experimental Section) so any band bending around GBs, e.g., as has been shown in graded devices previously,^[^ ^10^, ^44^ ^]^ becomes flattened and only intrinsic GI and GB resistivity are measured.

### 3.2 N‐Type Behavior

This section explores potential causes for the observed strong n‐type behavior and implications for device performance. Binary CdSe films are commonly n‐type,^[^ ^33^, ^34^ ^]^ so it was questioned whether n‐type behavior could originate from phase segregation into Se‐rich and Se‐poor regions, which would have wurtzite and zincblende structures, respectively. While the literature suggests this transition may happen at compositions as low as CdSe_0.3_Te_0.7_ in some cases,^[^ ^45^, ^46^ ^]^ X‐ray diffraction (XRD) analysis of the most Se‐rich films, CdSe_0.4_Te_0.6_, did not show evidence of the wurtzite phase here (Figure S8, Supporting Information). Some spatial variation in composition was observed in these films (Figure S9, Supporting Information), but it did not appear to affect the intrinsic electronic properties (Figure S2b, Supporting Information). Importantly, this shows that spatial nonuniformities in electrical properties seen in graded devices^[^ ^46^, ^47^ ^]^ are not inherent to polycrystalline CST thin films (here without intentional doping), but are likely driven by differences in composition within the stack (i.e., from sequential evaporation of CdSe or CST and CdTe followed by CdCl_2_ treatment, or co‐evaporation of CdSe and CdTe).

Several studies have shown n‐type GBs in CdTe^[^ ^44^, ^48^, ^49^ ^]^ and CST.^[^ ^10^, ^50^, ^51^ ^]^ It is possible that a common defect chemistry is shared between GBs and GIs, and as the density of GBs increases, the density of hole traps responsible for the trends seen in this work also increases. This may be why resistance (electron density) and grain size are particularly well correlated (Figure S7, Supporting Information). Thus, GBs contribute to, but are not solely responsible for, n‐type behavior in these films. By beveling the samples and measuring SSRM as a function of depth (Figure S3g, Supporting Information), it was shown that n‐type behavior was uniform throughout the film and not a result of altered chemistry at the front interface (e.g., oxidation,^[^ ^52^, ^53^ ^]^ accumulation of Cl/CdCl_2_).^[^ ^54^ ^]^ Hall effect measurements (not shown), which probe the bulk, also showed n‐type behavior.

Because most studies show slightly p‐type behavior in the bulk under standard device processing, particularly in CdTe, it is important to understand the conditions at which CST becomes strongly n‐type, and whether it commonly is in devices. This is particularly important since the conditions used here were similar to standard device processing and are standard conditions used for test structures. Jiang et al. previously showed n‐type behavior in the Se‐rich region of graded CST devices (not fabricated at NREL),^[^ ^24^ ^]^ which led to buried junction effects and associated losses. In a theoretical study by Good et al., a thin compensating layer at the front of GrV‐doped devices was shown to result in dramatic *V_OC_* loss.^[^ ^36^ ^]^ Generally, donor defects are undesirable since they can compensate p‐type dopants and/or compete for dopant sites (e.g., Cl or O competing with As for V_Te_ sites); see Section 3.3 for further discussion on potential defect chemistries.

High electron density (on the order of 10^16^ cm^−3^) in CST may also be a reason it is more difficult to dope p‐type than CdTe.^[^ ^55^ ^]^ This could also be a reason CST‐only devices (including graded CdSe_0.4_Te_0.6_/CdSe_0.2_Te_0.8_ devices) do not typically perform as well as graded CST/CdTe devices.^[^ ^56^ ^]^ In addition to the electron reflector role Shah et al. demonstrated CdTe plays at the back,^[^ ^56^ ^]^ recombination may be lower at the back of CST/CdTe devices due to, at least partially, the reduced electron density there. Knowing that CST (no intentional doping) can be strongly n‐type and accounting for this may open avenues to make highly doped n‐type devices, particularly since the CST measured here showed an electron density of 10^16^–10^17^ cm^−3^ (assuming an electron mobility of 100 cm^2^ Vs^−1^). Future work should evaluate whether CST in devices (which is typically treated at 450–480 ˚C CdCl_2_ rather than 500˚C since the latter typically causes delamination) has similarly high electron density. Finally, the inclusion of n‐type CST in device models, rather than assuming it is p‐type as is often done, may help elucidate differences between theoretical and measured device performance.

### 3.3 Potential n‐type Defect Chemistries

This section develops arguments for which defect chemistries are likely to contribute to the trends observed in this work, namely sub‐bandgap PL emission at room temperature and n‐type behavior. Polycrystalline CdTe films are typically slightly Te‐rich, and therefore p‐type, due to the lower formation energy of cation vacancies (V_Cd_).^[^ ^17^, ^57^ ^]^ Additional hole density is thought to originate from Cu impurities (on Cd sites) introduced during CdCl_2_ treatment.^[^ ^18^, ^58^ ^]^ For the films studied here to be n‐type (including CdTe), it is likely that a stable donor defect (or defects, defect complex(es)) has overwhelmed the intrinsic acceptor defects, potentially pinning the Fermi level and changing the conductivity. Since CdTe appeared mostly intrinsic with minimal n‐type behavior, it is likely that Se plays a key role in defect generation.

Anion vacancies (V_Se_, V_Te_) are a logical assumption since binary CdSe films are typically n‐type due to high densities of V_Se_,^[^ ^33^, ^34^ ^]^ and CdSe_x_Te_1‐x_ (specifically, CdSe_0.5_Te_0.5_) has been shown, theoretically, to have a lower formation energy for anion vacancies than CdTe.^[^ ^17^ ^]^ However, anion vacancies are not thought to be solely responsible for the sub‐bandgap defect emission and n‐type behavior observed here for a few reasons: i) the sample expected to have the highest anion vacancy density, “slow deposition CdSe_0.3_Te_0.7_,” which did have a lower measured Se % of CdSe_0.23_Te_0.77_, had higher resistance (lower electron density) and lower defect emission than baseline CdSe_0.3_Te_0.7_, ii) if CdSe_x_Te_1‐x_ has a higher density of isolated anion vacancies than CdTe, it would be expected to be easier to dope p‐type, but the opposite is typically observed,^[^ ^55^ ^]^ and iii) when chlorine is present, it is likely more thermodynamically favorable to form Cl_Te_ than V_Te_ in CdTe.^[^ ^59^ ^]^ It is possible, however, that the difference in DLTS between baseline and slow deposition CdSe_0.3_Te_0.7_, namely higher activation energy and the emergence of a deep electron trap in the latter, may be related to anion vacancies; further research is required.

Since all samples required CdCl_2_ treatment to be measurable via PL, TRPL, DLTS, etc., it is challenging to decouple Se from Cl effects. Cl_Te_ is a known shallow donor defect in CdTe and has been identified, primarily at GBs for polycrystalline material, in both CdTe^[^ ^44^, ^48^, ^49^ ^]^ and CST,^[^ ^10^, ^50^, ^51^ ^]^ turning them n‐type. Importantly, films that were not CdCl_2_ treated were too resistive to measure via Hall. Likewise, when the “CdCl_2_ anneal” was done without a CdCl_2_ source (i.e., CdSe_0.3_Te_0.7_ was annealed using the same temperature profile and ambient that might generate non‐Cl related defects, such as anion vacancies, Se_Te_, O_Te_), the films were too resistive to be measured. This suggests that the n‐type defects that dominate here are related to chlorine and/or require the presence of CdCl_2_ to be generated (e.g., impurities introduced from the CdCl_2_ source).

Importantly, the CdCl_2_ anneal has been identified as the primary driver for Se diffusion in CST.^[^ ^60^, ^61^, ^62^ ^]^ Because diffusion increases with temperature, it is possible that the more aggressive CdCl_2_ treatment used here (500 ˚C vs 450–480 ˚C commonly used in devices) moves Se around more, creating a higher probability for defect creation, whether it is impurity substitution (e.g., Cl_Te_, O_Te_), anti‐sites (e.g., Se_Te_), interstitials (e.g., Cd_i_), or vacancies (e.g., V_Se_). Interstitial chlorine may also exist at high chlorine concentrations (e.g., from long and/or high‐temperature CdCl_2_ anneals). If a high density of anion vacancies are generated during the CdCl_2_ anneal, Cd‐rich conditions would be created and the CdSe_x_Te_1‐x_ film may be easier to dope with Cl, as demonstrated in CdTe single crystal studies.^[^ ^19^, ^21^, ^49^ ^]^

Of course, defect complexes may also form, such as the chlorine “A‐center” (V_Cd_‐Cl_Te_), which acts as a shallow donor when in C_S_ symmetry.^[^ ^49^ ^]^ Oxygen complexes such as the oxygen “A‐center” (V_Cd_‐O_Te_) or Te_Cd_‐O_Te_ may also form, but these defects may tend to be shallow acceptors.^[^ ^63^, ^64^ ^]^ Divacancy complexes, e.g., V_Cd_‐V_Te_, are another possibility that has been identified in CdTe films via techniques like positron annihilation spectroscopy.^[^ ^65^, ^66^ ^]^ If this is the case, it would parallel the defect complex thought to be responsible for recombination and metastability in CuIn_x_Ga_1‐x_Se_2_: V_Se_‐V_Cu_.^[^ ^67^ ^]^ Further research is required to evaluate activation energy (e.g., as a function of Se content, CdCl_2_ anneal temperature, CdSe_x_Te_1‐x_ deposition rate at varied alloyed source compositions) to better understand which defect(s) can turn CdSe_x_Te_1‐x_ n‐type and which defects, if any, remain in GrV‐doped films. It would be enlightening to repeat density functional theory modeling^[^ ^68^ ^]^ with the inclusion of Cl, Se, and GrV dopants.

## 4 Conclusion

Se alloying of CdTe solar cells may present a double‐edged sword where the density of deep non‐radiative defects is reduced on one hand, but shallower, radiative donor defects are introduced on the other, which can turn the CdSe_x_Te_1‐x_ n‐type (with electron density on the order of 10^16^ cm^−3^) and limit *V_OC_*. Losses associated with n‐type CdSe_x_Te_1‐x_ (e.g., buried junction effects, compensation, low activation) may be amplified in highly‐doped GrV devices, which theoretical studies show are more sensitive to the front interface than low‐doped Cu devices. Additionally, CdSe_0.3_Te_0.7_ (the composition used in champion NREL‐grown devices) treated with GrV‐optimized CdCl_2_ conditions demonstrated increased nonradiative recombination, which may be one of the reasons GrV‐doped devices can have lower lifetimes than Cu‐doped devices.

Preferential loss of Se, as evidenced in the “slow deposition” sample which was evaporated from an alloyed CdSe_0.3_Te_0.7_ source but measured CdSe_0.23_Te_0.77_ in the final film, along with recent theoretical work suggests anion vacancies may be generated more readily in CdSe_x_Te_1‐x_ than CdTe. This may lead to a much higher density of anion vacancies, particularly during the CdCl_2_ anneal, which is the main driver for Se diffusion. This would then create optimal conditions for chlorine doping of CdSe_x_Te_1‐x_ (e.g., Cl_Te_ formation), which is of concern in GrV‐doped devices since dopants and Cl would compete for the same sites. This may be a reason that CdSe_x_Te_1‐x_ is harder to dope p‐type than CdTe. Importantly, this work suggests defect generation during the CdCl_2_ anneal may be more harmful than defects generated during CdSe_x_Te_1‐x_ deposition (e.g., anion vacancies in either case), meaning it may be more impactful to maintain a Se overpressure during the former. DLTS showed a band of defects, which may include defects that interact to form complexes.

Finally, the uniform n‐type behavior shown throughout the films here suggests that spatial non‐uniformities in electrical properties seen in graded samples, are not inherent in CdSe_x_Te_1‐x_, but are likely driven by differences in composition within the stack, so deposition from an alloyed source may be preferential. Understanding the conditions at which CdSe_x_Te_1‐x_ becomes n‐type and controlling/accounting for this behavior may help in realizing the full potential of Se alloying, both in standard p‐type CdTe architectures and potentially for highly doped n‐type CdSe_x_Te_1‐x_‐only devices.

## 5 Experimental Section

### Sample Preparation

CdSe_x_Te_1‐x_ test structures were fabricated on TEC12D, a commercial soda‐lime glass substrate coated with a conductive SnO_2_ layer. TEC12D substrates were used rather than uncoated glass (e.g., Eagle XG) to keep the structure of the CdSe_x_Te_1‐x_ (e.g., grain morphology, size, crystallinity) as similar to devices as possible. Test structure architecture varied slightly based on the characterization method. For TRPL and PL characterization, CdSe_x_Te_1‐x_ double heterostructures were fabricated by first depositing 100 nm of Al_2_O_3_ on the TEC12D surface via electron beam evaporation with no intentional heating of the substrate (pressure ≈mid 10^−6^ Torr, 2 Å/sec deposition rate). The 3 µm of uniform‐composition CdSe_x_Te_1‐x_ was then thermally evaporated from a ternary source powder (x = 0, 0.1, 0.2, 0.3, 0.4) at substrate temperature (T_sub_) = 450 ˚C. Typically, a deposition rate of 16 Å s^−1^ was maintained, except in the case that the deposition rate was intentionally decreased to 2 Å s^−1^ (“slow deposition” CdSe_0.3_Te_0.7_).

Film stacks were then annealed in a CdCl_2_‐rich ambient in a close‐space sublimation configuration with no Se overpressure. In most cases, the source material (CdCl_2_ beads) was held at T_source_ = 495 ˚C and T_sub_ = 500 ˚C for 10 min in 400 Torr He. In one case, CdSe_0.3_Te_0.7_ was annealed at T_sub_ = 500 ˚C with other conditions changed to what is commonly used for NREL‐grown GrV devices (labeled “GrV‐optimized CdCl_2_” in this work). Because this process uses proprietary conditions, they are not detailed here. After CdCl_2_ treatment, CdSe_x_Te_1‐x_ films were briefly rinsed in DI water, as is done in NREL‐grown devices. For PL and TRPL measurements, 20 nm of Al_2_O_3_ was evaporated (same conditions as above) followed by a second, lower‐temperature CdCl_2_ anneal (T_sub_ = 400 ˚C, 400 Torr He, 10 min). The wide bandgap Al_2_O_3_ layers that sandwich the CdSe_x_Te_1‐x_ are thought to provide field‐effect passivation and possibly also chemical passivation.^[^ ^52^ ^]^

For DLTS and SSRM measurements, CdSe_0.3_Te_0.7_ was grown directly on TEC12D rather than Al_2_O_3_‐coated TEC12D. CdCl_2_ was done as described above and 100 nm of Au was thermally evaporated on the back. A small amount of film stack was then scraped away to reveal the front contact. For SSRM measurements, these film stacks were thermo‐mechanically cleaved at the SnO_2_/CdSe_x_Te_1‐x_ interface using a process similar to that described by Perkins et al.^[^ ^54^ ^]^ Briefly, an Al shim handle was epoxied to the CdSe_x_Te_1‐x_ back surface with a conductive Ag‐filled epoxy (Epo‐tek H20E) and annealed in an oven overnight at 80 ˚C. These stacks were then dipped into liquid nitrogen (LN_2_) within an Ar‐filled glovebox until spontaneous cleavage occurred. For AES and Hall, CdCl_2_‐treated CdSe_x_Te_1‐x_ films grown on Al_2_O_3_‐coated TEC12D (no back surface layers) were cleaved using the same process, where samples for Hall were cleaved using an insulating epoxy (Hysol 1C). After cleavage, the CdSe_x_Te_1‐x_ side of the cleave was extracted from the LN_2_ bath into a stream of dry N_2_ until room temperature was reached. Samples were then transferred without air exposure into the respective characterization tool for measurement.

### Characterization

TRPL measurements were taken on a home‐built system described elsewhere.^[^ ^41^ ^]^ A diode laser with 670 nm wavelength excitation and 50 µm beam diameter was used at a repetition rate of 125 kHz. Laser power measured at the sample was 0.11 µW, giving a fluence of ∼2 × 10^11^ cm^−2^ and injection level of ∼8 × 10^15^ cm^−3^ assuming a generation depth of 200 nm for 670 nm excitation in CdSe_0.3_Te_0.7_.^[^ ^41^ ^]^ A longpass filter of 700 nm (∼1.77 eV) was placed in the optical path before the Si avalanche photodiode detector.

PL measurements were taken using 632.8 nm excitation with a HeNe laser of beam diameter 0.9 mm at 1 Sun equivalent excitation (2 × 10^21^ photons/(m^2^s)). A pairing of spectrally corrected Si and InGaAs detectors (PIX100F Si CCD and Pylon IR, respectively) was used to obtain a larger spectral range (Si sensitive up to ≈960 nm, InGaAs sensitive at longer wavelengths, see Figure S10, Supporting Information). Detectors were calibrated using an IntelliCal intensity calibration system (Princeton Instruments). A comparison with absolute reflectance standards (LabSphere) was used to measure PL emission spectra in absolute photon numbers.

Prior to AES, CdSe_x_Te_1‐x_ films were thermo‐mechanically cleaved from their TEC12D substrates using the process described above. To better understand bulk composition, exposed films were first sputtered with a 2 kV ion beam at 70° angle measured from the surface normal and while rotating at 1 rpm. AES sensitivity factors were calculated as described previously^[^ ^69^ ^]^ using sputter depth profile data on an ungraded CdSe_0.08_Te_0.92_ film whose composition had been determined by X‐ray fluorescence. AES measurements were done using a 5 kV, 20 nA beam. The spectrometer binding energy scale was calibrated at high and low energy using clean gold and copper foils and known transition energies.^[^ ^70^ ^]^ Data analysis and peak fitting were performed using a combination of Igor and PHI MultiPak.

DLTS data were collected using a SULA Technologies digital model DDS‐12 DLTS system.^[^ ^71^ ^]^ This system uses a 1 MHz modulating signal. The samples were measured between 0.3 V reverse bias and 0 V. Capacitance transients were averaged with a 40 s time constant with temperature held steady during measurement of all transients in the designated time windows (2, 5, 10, 20, 50, and 100 ms).

SSRM was done using an AFM (Veeco D5000 and Nanoscope V) housed in an Ar‐filled glovebox. During measurement, a highly doped (p‐type) diamond‐coated Si probe (Bruker‐nano DDESP) is pressed into the sample with a large indentation force (≈µN), and a large bias voltage (≈5 V) is applied at the back contact. The total resistance (*R*_tot_) measured is composed of the spreading resistance (*R*_sp_) of the sample, contact resistance at the probe/sample interface (*R*_c_), and back‐contact resistance (*R*_b_). R_b_ is much smaller than *R*_c_ and *R*_sp_ since current pathways are spread out when reaching the back contact and series resistance in the film is relatively low (typically only a few Ω · cm^2^). Thus, if contact resistance is minimized (by using a large indentation force and large forward bias voltage and maintaining an inert ambient to prevent sample oxidation),^[^ ^22^, ^72^ ^]^ the measured resistance is dominated by *R*_sp_. For depth profiling, samples were bevel‐polished from the back using plane‐view ion‐milling at a maximum glancing angle of 7˚.

Hall measurements were done using an Accent HL5500PC system. Thermomechanical cleaving, as described above, results in films with irregularly shaped areas so prior to measurement, a square ≈5 × 5 mm was cut through the film and epoxy. Indium contacts were placed in the corners and the van der Pauw technique was used. XRD measurements were made using a Rigaku DMAX X‐ray diffractometer that was set up using Bragg‐Brentano geometry. A Cu Kα radiation source was used at 40 kV and 250 mA excitation, and samples were scanned from 20 to 140 degrees 2θ. Phase information and lattice parameters for CdSe_x_Te_1‐x_ were extracted from the literature.^[^ ^45^ ^]^

Optical images were taken on CdCl_2_‐treated CdSe_x_Te_1‐x_ (no back surface layers) using a Zeiss M2m Imager with AxioVision software at 100x magnification. While a Benson etch^[^ ^73^ ^]^ is typically required to increase the contrast between grain boundaries and interiors (GBs are preferentially etched), this was not required here. Average grain size was calculated using ImageJ software and standard E112‐12 developed by the American Society for Testing and Materials (ASTM).^[^ ^74^ ^]^

## Conflict of Interest

The authors declare no conflict of interest.

## Supporting information

Supporting Information

## Data Availability

The data that support the findings of this study are available from the corresponding author upon reasonable request.
