# Supplementary material for: Investigation of Sub‐Bandgap Emission and Unexpected n‐Type Behavior in Undoped Polycrystalline CdSexTe1‐x
Source: Adv Sci (Weinh). 2024 Jun 3;11(29):2309264. doi: 10.1002/advs.202309264 (PMC11304320; doi:10.1002/advs.202309264)
Supplement: Supplementary file 1 — Supporting Information [file ADVS-11-2309264-s001.docx]

**Supplemental Information**

**Investigation of sub-bandgap emission and unexpected n-type behavior in undoped polycrystalline CdSe_x_Te_1-x_**

Deborah L. McGott, Steven W. Johnston, Chun-Sheng Jiang, Tuo Liu, Darius Kuciauskas, Stephen Glynn, Matthew O. Reese*

**Corresponding author:** Matthew O. Reese

National Renewable Energy Laboratory, Golden Colorado 80401, United States

Matthew.Reese@nrel.gov


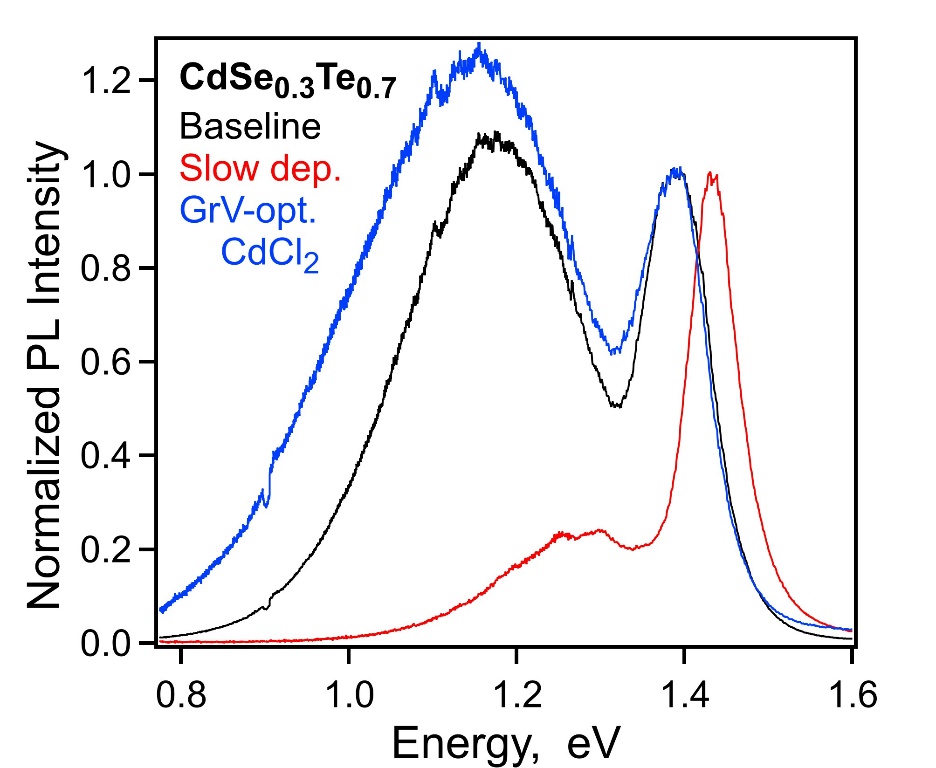


**Figure S1.** Spectrally corrected normalized PL data for CdSe_0.3_Te_0.7_ with baseline conditions (black), slow deposition (red), and treated with CdCl_2_ anneal conditions optimized for NREL-grown GrV doped devices (blue). CdSe_0.3_Te_0.7_ refers to the alloyed source material composition, not measured Se % in film. Absolute PL data shown in Figure 1b in main text.

**
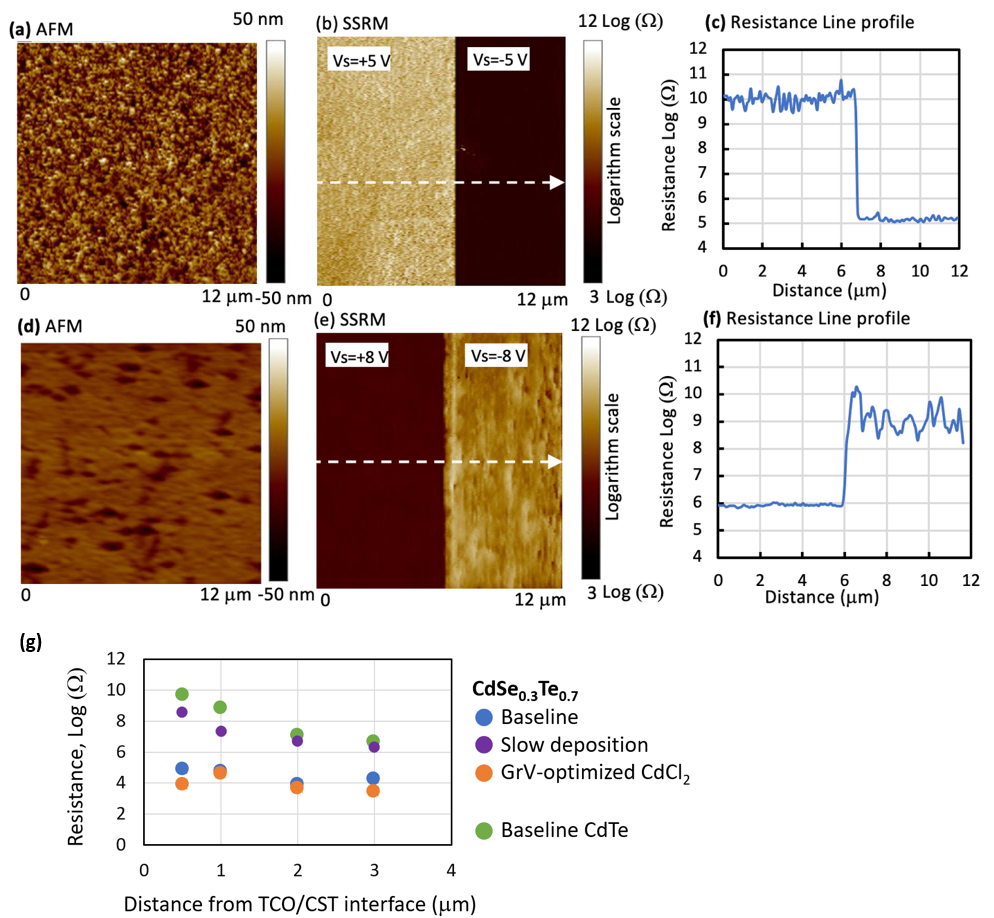
**

**Figure S2.** Example (a) AFM and (b) corresponding SSRM for CST (CdSe_0.2_Te_0.8_ in this case) with applied bias +5 V and -5 V (light left and dark right sides, respectively). (c) shows resistance profile taken along the dashed arrow shown in (b), demonstrating high resistance under positive bias and low resistance under negative bias, indicating an n-type material. (d)-(f) show similar maps for a known p-type material (As-doped CdTe grown via molecular beam epitaxy with 3.5x10^16^/cm^3^ carrier concentration measured via Hall effect), demonstrating opposite behavior to what is seen in (b)-(c) by flipping the bias voltage polarity. (g) shows average resistance as a function of depth for bevel polished samples (CdSe_0.3_Te_0.7_ series – baseline, slow deposition, and grV-optimized CdCl_2_ – and baseline CdTe for reference). Increasing resistance toward the front interface for CdTe and slow deposition CdSe_0.3_Te_0.7_ is likely due to increased influence of the junction for this low-doped material.


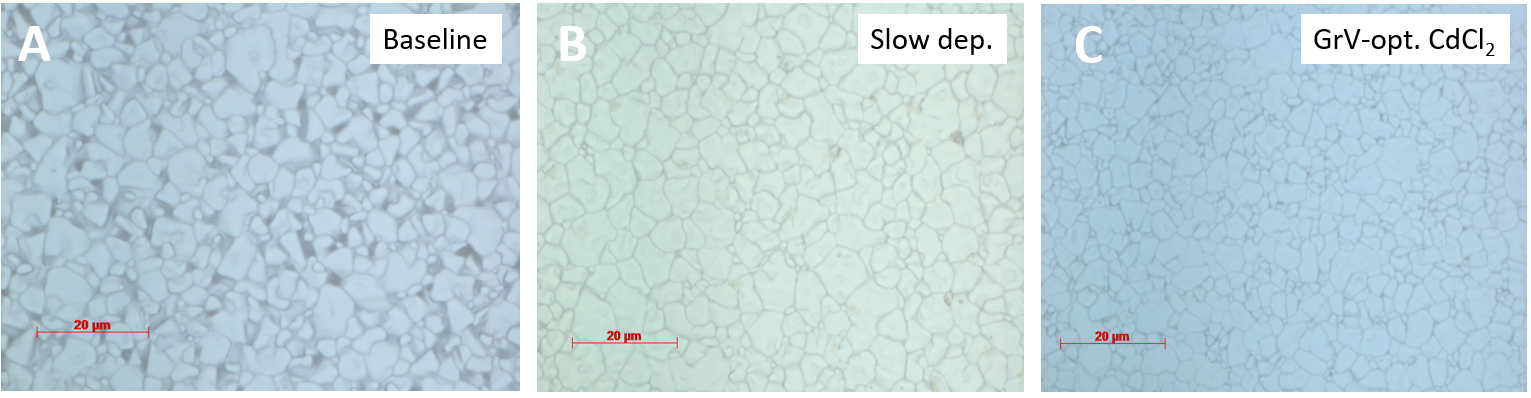


**Figure S3.** Optical microscope images of films deposited from CdSe_0.3_Te_0.7_ alloyed source with (a) baseline conditions, (b) slow deposition, and (c) CdCl_2_ anneal conditions optimized for NREL-grown GrV doped devices.


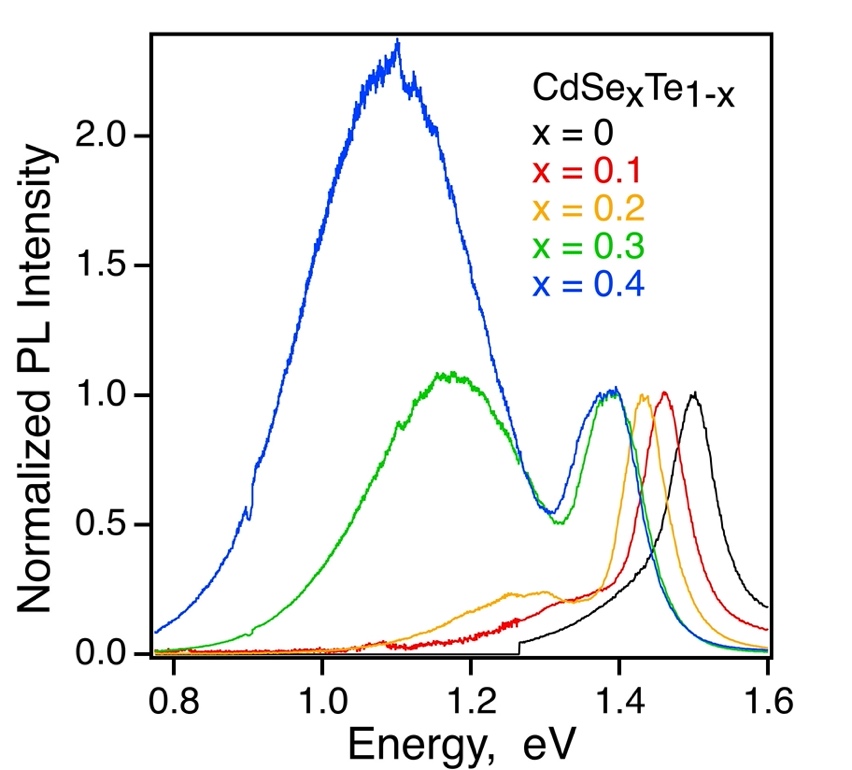


**Figure S4.** Spectrally corrected normalized PL for CdSe_x_Te_1-x_ with x = 0, 0.1, 0.2, 0.3, and 0.4 (referring to the alloyed source material composition) with baseline conditions; absolute PL data shown in Figure 3b in main text.


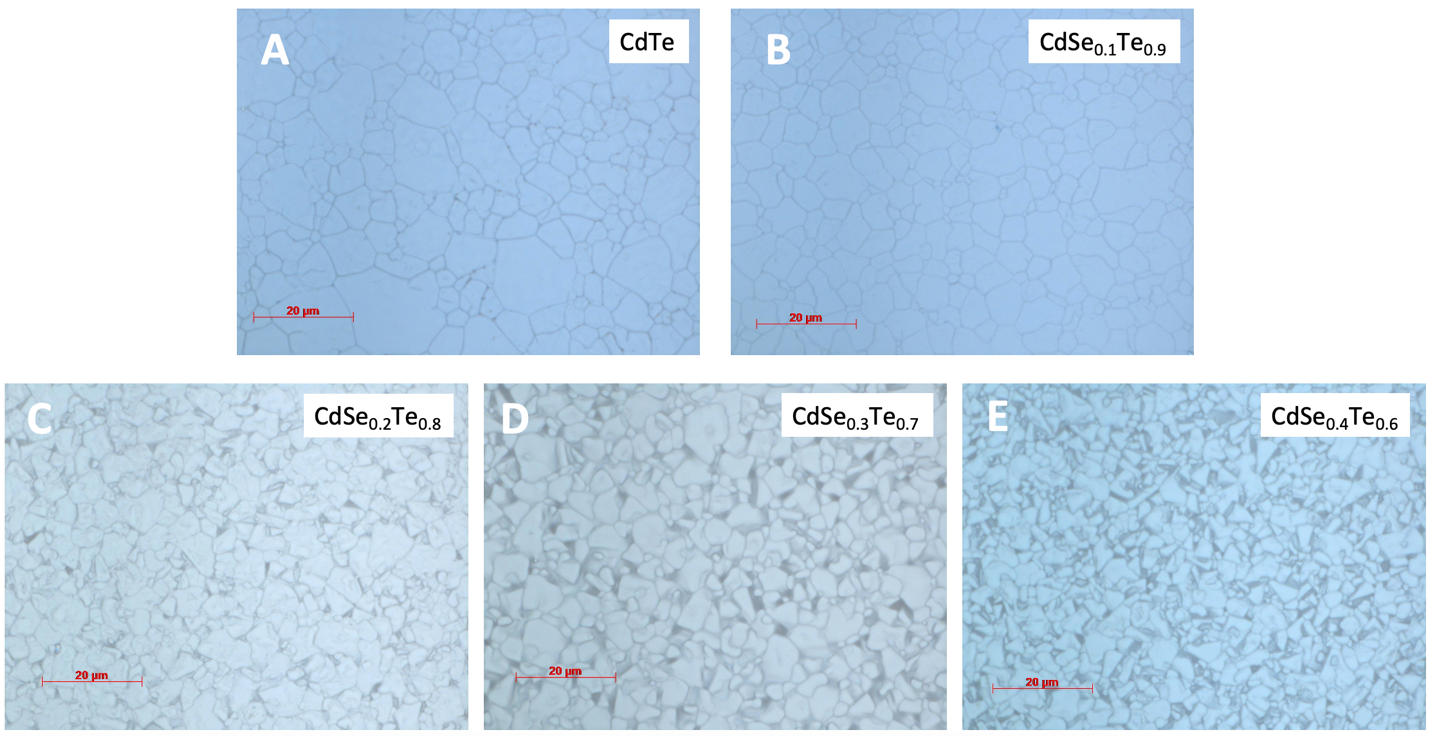


**Figure S5.** Optical microscope images of CdSe_x_Te_1-x_ films with (a) x = 0, (b) x = 0.1, (c) x = 0.2, (d) x = 0.3, and (e) x = 0.4 (referring to the alloyed source material composition) with baseline conditions, showing decreasing grain size as Se % increases.


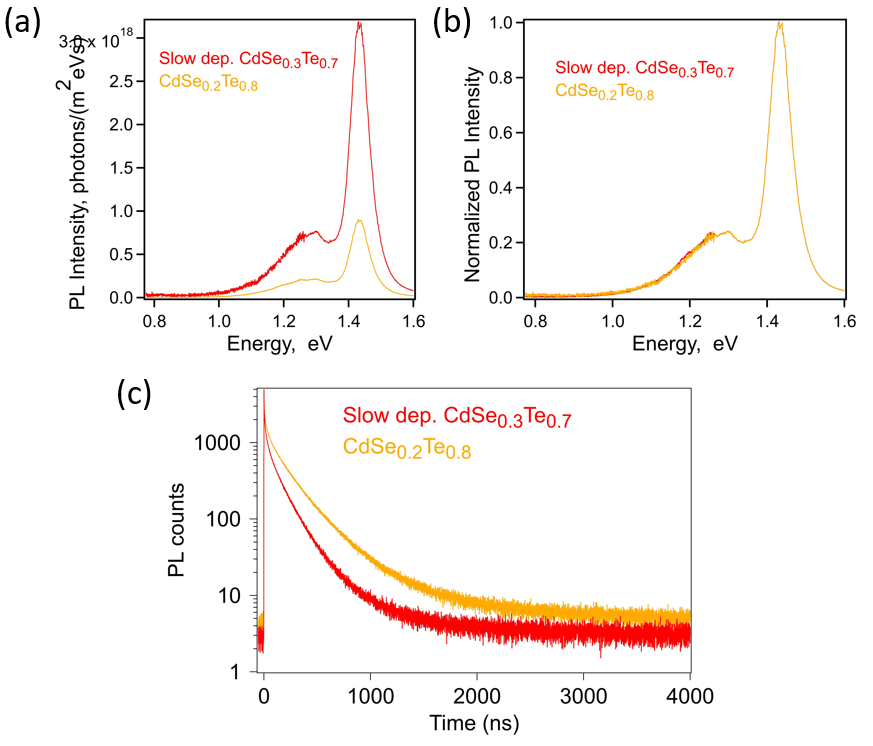


**Figure S6.** (a) absolute and (b) normalized spectrally corrected PL, (c) TRPL of “slow deposition CdSe_0.3_Te_0.7_” (referring to the composition of the alloyed source powder; measured composition in film was CdSe_0.23_Te_0.77_) compared to CdSe_0.2_Te_0.8_ with baseline conditions (measured CdSe_0.19_Te_0.81_ in film). The similarity in normalized PL despite different measured Se % in the films may be a result of spatial non-uniformity (PL measurement diameter is ~1 mm, AES measurement area is 33 $\mu$m x 33 $\mu$m), the fact that both compositions are near the bottom of the bandgap bowing curve,^1^ and/or differences in measurement resolution between AES and PL.

**Table S1.** Measured parameters “slow deposition CdSe_0.3_Te_0.7_” (referring to the composition of the alloyed source powder; measured composition in film was CdSe_0.23_Te_0.77_) compared to CdSe_0.2_Te_0.8_ with baseline conditions. $\tau$_2_ was measured via TRPL; *E_exciton_*, *E_defect_*, and PLQY via PL; Se/(Se+Te) measured in the final films via AES; R_tot_ measured using SSRM; and grain size measured with bright-field optical microscope.

|  | $\boldsymbol{\tau}$**_2_**  **(ns)** | **E_exciton_ (eV)** | **E_defect_**  **(eV)** | **PLQY** | **Se/**  **(Se+Te)** | **R_tot_**  **(**$\boldsymbol{\Omega}$**)** | **Avg. grain size**  **(µm)** |
| --- | --- | --- | --- | --- | --- | --- | --- |
| **Slow dep. CdSe_0.3_Te_0.7_** | 150 | 1.43 | 1.30 | 2.2E-04 | 23 | 3.7E+05 | 2.2 ± 0.3 |
| **Baseline CdSe_0.2_Te_0.8_** | 230 | 1.43 | 1.28 | 6.2E-05 | 19 | 1.8E+05 | 2.3 ± 0.2 |

**
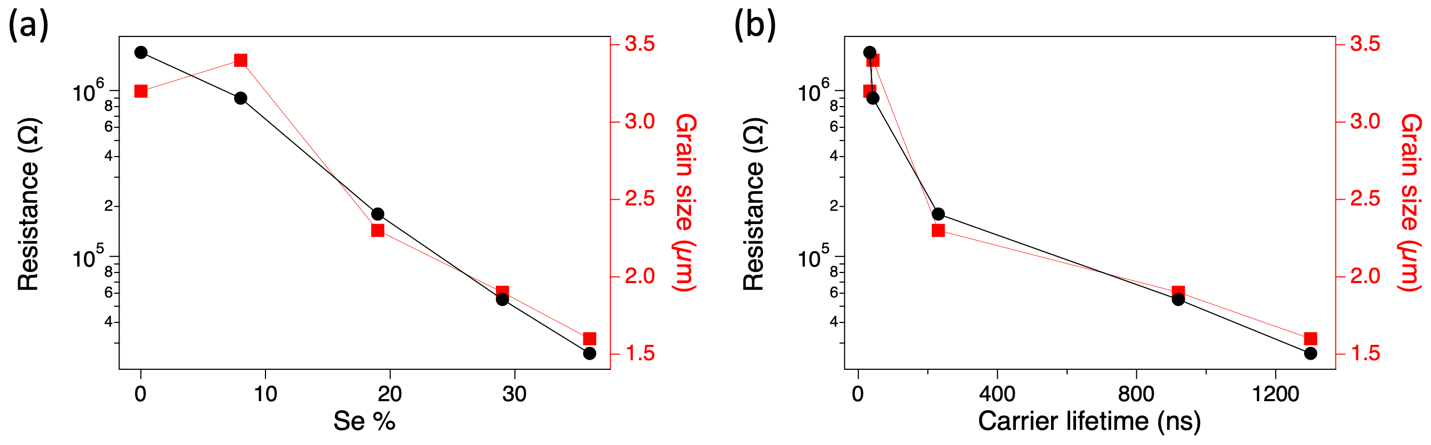
Figure S7.** Plots of SSRM-measured average resistance (black circles) and average grain size (red squares) as a function of (a) AES-measured Se % and (b) TRPL-measured minority carrier lifetime ($\tau$_2_) for films deposited from CdSe_x_Te_1-x_ (x = 0, 0.1, 0.2, 0.3, 0.4) alloyed source powder with baseline conditions.


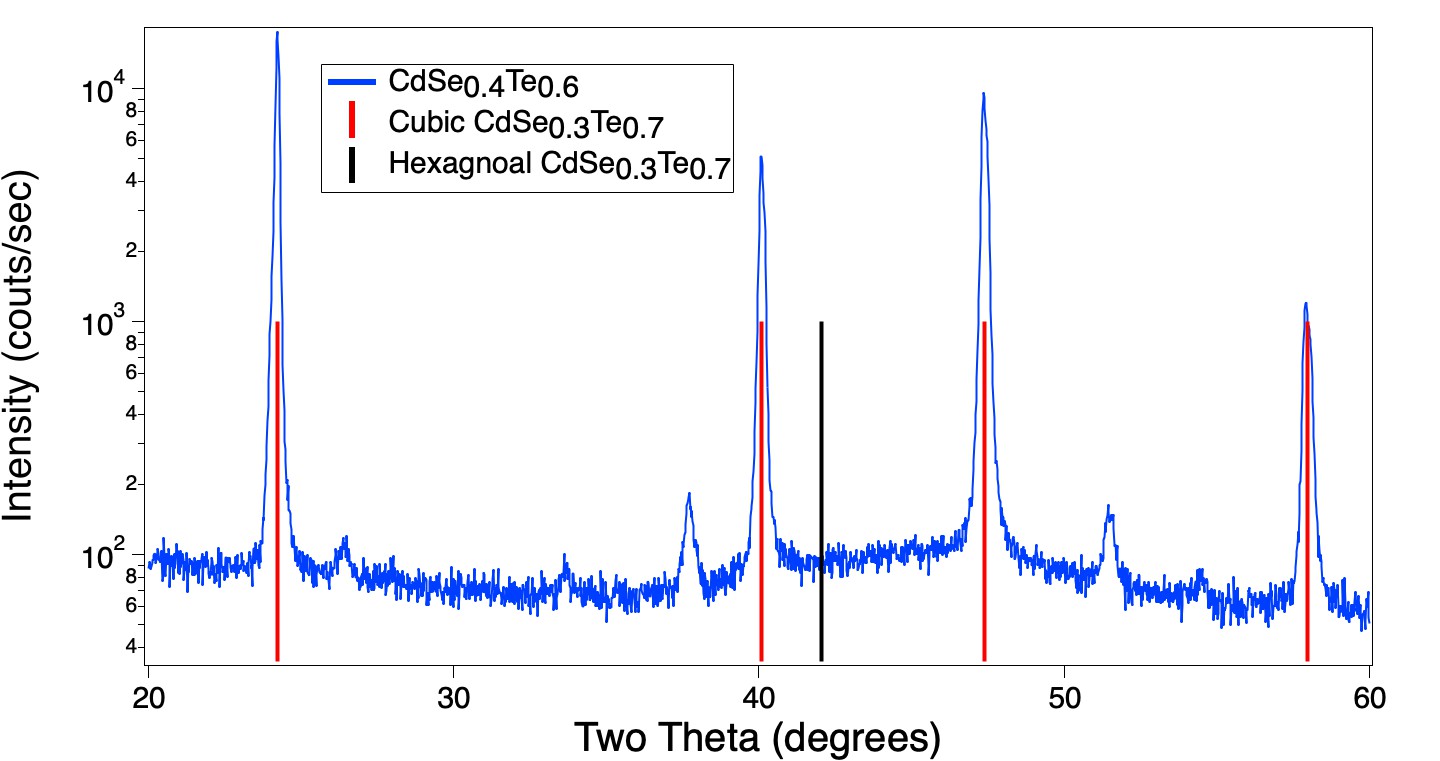


**Figure S8.** XRD spectrum of CdSe_0.4_Te_0.6_ film with two theta of cubic and hexagonal (wurtzite) phases for CdSe_0.3_Te_0.7_ labeled. Note: While the peaks lined up best with CdSe_0.3_Te_0.7_, this does not imply that Se concentration is actually lower than what was measured with AES. Many factors can influence spectra shift in XRD including strain, presence of other layers, etc.


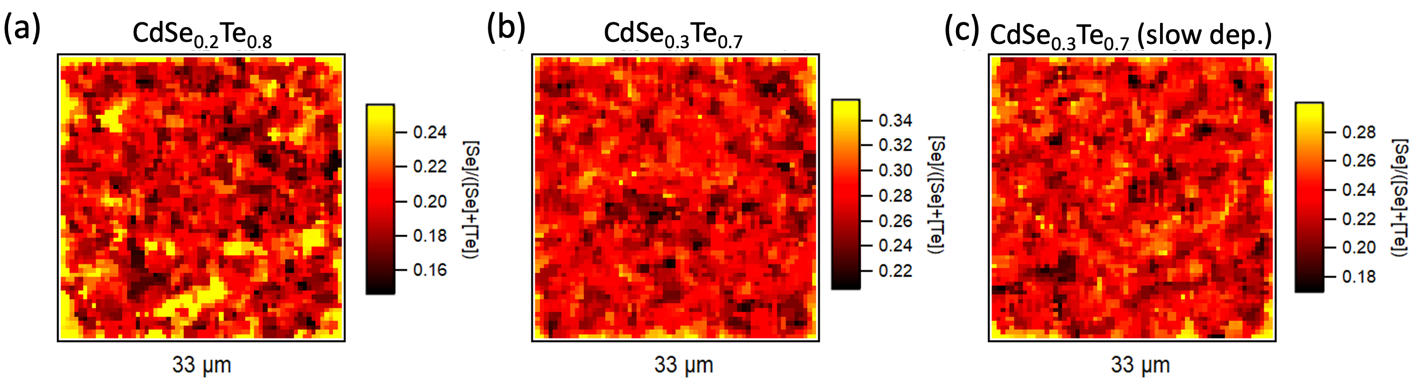


**Figure S9.** Example AES maps showing non-uniformities in Se % for (a) baseline CdSe_0.2_Te_0.8_, (b) baseline CdSe_0.3_Te_0.7_, and (c) “slow dep.” CdSe_0.3_Te_0.7_ (measured CdSe_0.23_Te_0.77_).


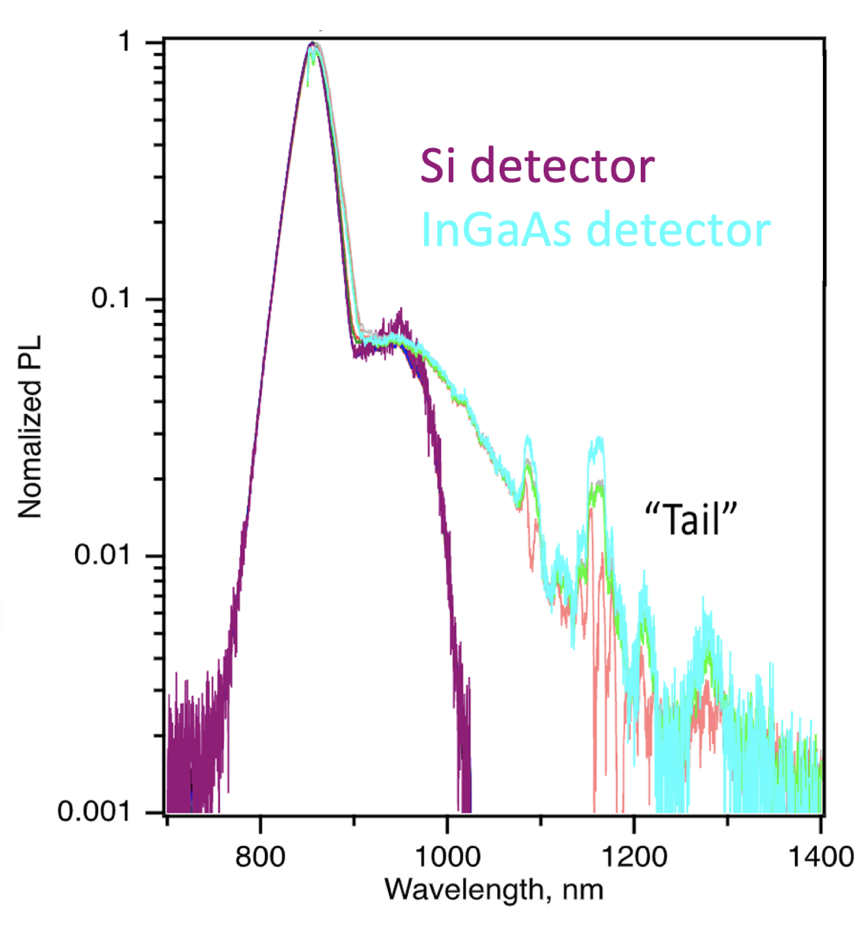


**Figure S10.** Normalized room-temperature spectral PL showing the difference between using a Si CCD detector which is sensitive up to ~950 nm (purple) and InGaAs detector which is sensitive from ~850 nm (teal, pink, lime green). A combination of spectrally corrected Si and InGaAs detectors was used in this work.

**References**

(1) Lane, D. A review of the optical band gap of thin film CdSxTe1− x. *Solar Energy Materials and Solar Cells* **2006**, *90* (9), 1169-1175.
